# Genetic structure and ecogeographical adaptation in wild barley (*Hordeum chilense *Roemer et Schultes) as revealed by microsatellite markers

**DOI:** 10.1186/1471-2229-10-266

**Published:** 2010-11-30

**Authors:** Almudena Castillo, Gabriel Dorado, Catherine Feuillet, Pierre Sourdille, Pilar Hernandez

**Affiliations:** 1 Instituto de Agricultura Sostenible (IAS, CSIC), Alameda del Obispo s/n, 14080 Córdoba, Spain; 2 Dep. Bioquímica y Biología Molecular, Campus Rabanales, C6-1-E17, Universidad de Córdoba, 14071 Córdoba, Spain; 3 INRA UBP UMR 1095, Genetics, Diversity & Ecophysiology of Cereals, Clermont Ferrand, France

## Abstract

**Background:**

Multi-allelic microsatellite markers have become the markers of choice for the determination of genetic structure in plants. Synteny across cereals has allowed the cross-species and cross-genera transferability of SSR markers, which constitute a valuable and cost-effective tool for the genetic analysis and marker-assisted introgression of wild related species. *Hordeum chilense *is one of the wild relatives with a high potential for cereal breeding, due to its high crossability (both interspecies and intergenera) and polymorphism for adaptation traits. In order to analyze the genetic structure and ecogeographical adaptation of this wild species, it is necessary to increase the number of polymorphic markers currently available for the species. In this work, the possibility of using syntenic wheat SSRs as a new source of markers for this purpose has been explored.

**Results:**

From the 98 wheat EST-SSR markers tested for transferability and polymorphism in the wild barley genome, 53 primer pairs (54.0%) gave cross-species transferability and 20 primer pairs (20.4%) showed polymorphism. The latter were used for further analysis in the *H. chilense *germplasm. The *H. chilense*-*Triticum aestivum *addition lines were used to test the chromosomal location of the new polymorphic microsatellite markers. The genetic structure and diversity was investigated in a collection of 94 *H. chilense *accessions, using a set of 49 SSR markers distributed across the seven chromosomes. Microsatellite markers showed a total of 351 alleles over all loci. The number of alleles per locus ranged from two to 27, with a mean of 7.2 alleles per locus and a mean Polymorphic Information Content (PIC) of 0.5.

**Conclusions:**

According to the results, the germplasm can be divided into two groups, with morphological and ecophysiological characteristics being key determinants of the population structure. Geographic and ecological structuring was also revealed in the analyzed germplasm. A significant correlation between geographical and genetic distance was detected in the Central Chilean region for the first time in the species. In addition, significant ecological influence in genetic distance has been detected for one of the population structure groups (group II) in the Central Chilean region. Finally, the association of the SSR markers with ecogeographical variables was investigated and one marker was found significantly associated with precipitation. These findings have a potential application in cereal breeding.

## Background

Wild species usually exhibit large genetic variability, which serves as a resource for adaptability to changing environments. On the contrary, cultivated plants are usually more limited in number and display less genetic variability, as a result of the genetic bottlenecks occurring at domestication, translocation and transition from landraces to modern breeding [1]. A consequence of such genetic erosion is genetic uniformity, which may result in the loss of relevant traits, such as resistance to biotic and abiotic stresses.

Thus, wild species related to cultivated crops represent interesting sources of genetic variation, through the introgression of new and better performing alleles. The large genetic variability present in the wild cereals is an invaluable resource for cereal crop improvement. *Hordeum chilense *Roemer et Schultes, a native South American diploid wild barley (2n = 2x = 14), offers a high potential for cereal breeding among the species of the genus *Hordeum*, because of its high crossability with other members of the Triticeae tribe and its agronomically interesting characteristics. Crosses between wheat and *H. chilense *lead to fertile amphiploids named tritordeums. They represent the basic genetic material for introducing genetic variability from *H. chilense *into wheat breeding programs [2] and for transferring useful genes from *H. chilense *to wheat. The analysis of the germplasm genetic structure is the basis of management, research and utilization of such germplasm [3], since it is critical to identify and correctly interpret the associations between functional and molecular diversity [4,5]. *H. chilense *has been found in a wide range of environments, and shows high genetic as well as phenotypic diversity [6]. The analysis of the structure of such high variations is important for breeding purposes, especially to identify genes or genomic regions involved in environmental adaptation and showing high diversity. The genetic structure of populations has been widely documented in most of the studies investigating the diversity of elite crop germplasm, especially in self-pollinating cereals [7-9]. Molecular markers and development of statistical techniques to analyze such data have been the subject of recent intensive studies [10-21], allowing the analysis of the genetic structure in several species and eliminating many of the problems linked with spurious associations. However, although significant efforts to increase the availability of genomic tools, such as molecular markers for cereal crops, have been undertaken in the last years, these developments were not made for wild species with scarce direct agronomic interest, like *H. chilense*. To solve this problem, the transferability of wheat and barley microsatellite markers (or Simple Sequence Repeats; SSR) to wild related species was evaluated [22,23]. Comparative genomic analyses have indicated a good conservation of coding regions across genomes of different grass species, suggesting that this part of the genome can be used to develop transferable molecular markers [24-27]. The development of high throughput sequencing technologies in recent years has allowed the generation of large Expressed Sequence Tag (EST) datasets in a number of plant species, including cereals, which can be systematically searched for SSR [28]. For example, Yu et al. [29] tested EST-SSR primers originating from hexaploid wheat and rice ESTs on four cereal crops (wheat, rice, barley and maize) and found that 62% of the primer pairs produced Polymerase Chain Reaction (PCR) amplicons on at least two species. Similarly, Zhang et al. [30] reported the transferability of 116 wheat EST-SSRs on 168 accessions, representing 18 grass species. The transferability among the Triticeae ranged from 73.7% for *Aegilops longissima *to 100% for wheat subspecies (*Triticum compactum*), but was also good for less related species such as rye (72.8%) or maize (40.4%). In barley, Varshney et al. [31] reported that 78.2% of the SSR markers used (165) showed amplification in wheat, followed by 75.2% in rye and 42.4% in rice. Finally, Gupta et al. [32] reported that 55.12% of wheat EST-SSRs were transferable to barley. Recently, it was shown that the barley EST-SSRs represent a promising source of molecular markers to screen the *H. chilense *genome [33].

In addition of their high degree of transferability across species, it was recently demonstrated that EST-SSRs are useful for genetic variability studies. For example, Gupta et al. [32] assessed the genetic diversity of EST-SSRs in a collection of 52 elite exotic wheat genotypes. Their results indicate that EST-SSRs are more useful for diversity analyses than genomic microsatellites (g-SSRs). Yang et al. [34] also used EST-SSRs to measure the genetic diversity among three hexaploid wheat populations. They concluded that EST-SSR markers are ideal markers for assessing genetic diversity in wheat. In addition, Balfourier et al. [35] used 38 g-SSRs or 44 EST-SSRs to analyze the structure and the diversity of a collection of 372 wheat varieties and obtained identical results with both types of markers. Recently, Hübner et al. [21] studied the genetic analysis of a new collection of the wild barley *H. spontaneum *with a set of 42 EST-SSRs, revealing that wild barley populations can be divided into seven major genetically differentiated clusters, as well as the evidence of temperature and precipitation as environmental cues that have shaped the genetic makeup of wild barley. Pan et al. [36] investigated the genetic diversity among 15 wild emmer wheat (*T. dicoccum*) populations using 25 EST-SSRs, detecting a considerable amount of genetic variation, partly related to ecological factors.

The goals of the current study were to determine: (i) the transferability level of wheat EST-SSR markers and their usefulness for *H. chilense*; (ii) the genetic diversity of the *H. chilense *species, using a wide set of available microsatellite markers; (iii) the genetic structure of a natural collection of 94 *H. chilense *accessions; and (iv) the possible influence of spatial, morphological and environmental factors in the observed structure.

## Results

### Transferability and polymorphism of wheat EST-SSRs

The transferability of the 98 wheat EST-SSRs was evaluated on a set of eight accessions of *H. chilense*. A SSR was considered as transferable when the PCR amplification of a band of the expected size and SSR pattern was observed. The primers that showed null alleles in some samples were tested at least twice, in order to avoid false negatives (eg., non-amplification due to PCR failure).

Among the 98 wheat EST-SSRs, 53 (54%) showed cross-species transferability. The percentage of transferred markers was about 50% on each chromosome, thereby indicating a uniform distribution across the genome. Among the 53 transferable SSRs, 20 PCR primer pairs (20.4%) showed polymorphism in the accessions studied and were used for further analysis in the *H. chilense *germplasm. Between two and 10 alleles per primer pair were observed and only 11.7% of the total alleles had the same size as the allele found in *Triticum aestivum *cv. 'Chinese Spring', confirming the good potential of *H*. chilense for wheat and barley genetic diversity improvement. These markers were first assigned to *H. chilense *chromosomes using the available set of wheat-*H. chilense *addition lines ([37]). Fifteen of the 20 polymorphic primer pairs were located on the same linkage group as in wheat. One of them was located on chromosome 7 D in wheat (CFE135), while it amplified a product on chromosome 1H^ch ^in *H. chilense*. Four markers showed the same PCR amplicon sizes in both species, and thus their locations could not be confirmed.

### Genetic variability analysis

To perform the genetic variability analysis, 21 barley EST-SSRs and 8 gSSRs previously identified as useful for the genetic analysis in *H. chilense *([33,38]) were added to the 20 polymorphic wheat EST-SSRs transferred in this work. A total of 351 alleles were detected over the whole sample of 94 accessions for the 49 SSR loci. Among the 351 alleles, 162 originated from the 21 barley EST-SSRs, 94 from the 20 wheat EST-SSRs and 95 from the eight g-SSRs. The number of alleles per locus ranged from two (for GBM1411, GBM1323, GPW7425, CFE10 and CFE23) to 27 (for GBM1464), with a mean of 7.2 alleles and Polymorphic Information Content (PIC) of 0.5 per locus (Table 1). The highest (0.91) and lowest (0.04) PIC values were observed for GBM1464 and GPW7213, respectively. Generally, wheat EST-SSRs exhibited lower PIC values and fewer number of alleles than barley EST-SSRs. Barley EST-SSRs detected almost twice more alleles and higher PIC values than wheat EST-SSRs.

**Table 1:** Summary of the genetic parameters shown by 49 SSR markers used for the characterization of the H. chilense accessions.

Marker ID	Chromosome location	**Allele No**.	PIC
GPW7577	1B	5	0.140
CFE023	1B	2	0.331
GPW7296	1D	3	0.189
GBM1029	1H	7	0.606
GBM1002	1H	5	0.574
GBM1411	1H	2	0.369
GBMS14	1H	14	0.832
CFE175	2A	3	0.177
GPW7438	2B	6	0.153
CFE068	2D	7	0.679
GBM1047	2H	4	0.396
GBM1036	2H	7	0.515
GBM1462	2H	6	0.673
GBMS233	2H	13	0.736
GPW7213	3A	3	0.042
GPW7335	3B	8	0.581
GWM1047	3D	19	0.883
GPW7553	3D	8	0.700
GPW7663	3D	6	0.144
GBM1069	3H	6	0.605
GBMS198	3H	8	0.633
CFE188	4B	3	0.193
GWM1302	4D	10	0.800
GBM1055	4H	8	0.627
GBM1067	4H	9	0.485
GBM1020	4H	6	0.638
GBM1465	4H	5	0.222
GBM1323	4H	2	0.361
GBM1350	4H	19	0.906
GBMS214	4H	3	0.402
CFE037	5A	4	0.093
GPW7425	5B	2	0.078
CFE239	5B, 5D	3	0.072
GBM1064	5H	5	0.603
GBMS154	5H	10	0.674
GPW7455	6A	6	0.511
CFE002	6A, 6B, 6D	8	0.497
CFE080	6A, 6B, 6D	2	0.172
GWM1103	6D	18	0.873
GBM1008	6H	13	0.744
GBM1076	6H	6	0.701
GBM1400	6H	3	0.557
CFE010	7A, 7B, 7D	2	0.198
CFE100	7A, 7B, 7D	10	0.820
CFE135	7D	3	0.390
GBM1060	7H	13	0.838
GBM1058	7H	3	0.217
GBM1464	7H	27	0.913
GBM1432	7H	6	0.724

Mean		7.163	0.4951

Additionally, several rare or specific alleles were found among the analyzed germplasm. Out of the 351 alleles, 184 were found at a frequency lower than 5%, and were therefore considered as rare. Clustering showed that the germplasm can be separated into two groups (see below) with a total of 66 specific alleles found in group I (39 being rare) and 134 specific alleles found in group II (82 being rare). The gene diversity, polymorphic information content (PIC), and the number of specific alleles was lower in group I than in group II (Table 2).

**Table 2:** Number of accessions and specific alleles, gene diversity and its standard deviation (SD), calculated for 49 microsatellite loci typed for the H. chilense germplasm, per genetic structure group.

Structure groups	No. of accessions	No. of specific alleles	Gene diversity	Gene diversity SD
**Group I**	41	66	0.3811	0.0428

**Group II**	53	134	0.4874	0.0387

### Analysis of genetic structure and differentiation among inferred groups

**Figure 1:**
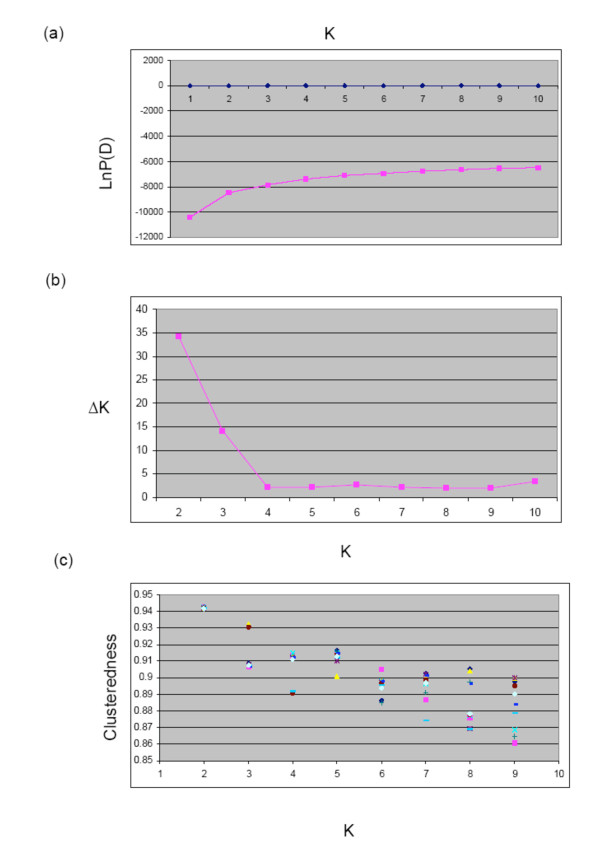
**Estimation of the most probable number of clusters (K), based on 20 independent runs and K ranging from 1 to 10**. (a) Evolution of the natural logarithm probability of the data against K; (b) magnitude of ΔK for each K value; and (c) clusteredness analysis of the *H. chilense *accessions.

The genotyping data obtained from the 49 SSRs were used to analyze the genetic structure of the germplasm, using the Bayesian clustering model implemented in the Structure software. The natural logarithm of the probability of the data, proportional to the posterior probability of K, showed no clear peak between 1 and 10 for K, and therefore it was difficult to determine the true number of populations (K) (Figure 1a). We applied the rate of change in the Napierian logarithm probability relative to standard deviation (ΔK), as described by Evanno et al. [39]. The results showed the highest peak at 2 (Figure 1b), which was confirmed by the clusteredness index [40], showing the highest median level at K = 2 (Figure 1c). Moreover, using the Geneland software, we observed that the posterior distributions of the estimated number of populations (K) across 10 replicates displayed a clear mode at K = 2 in all of them (additional file 1), corroborating again the previous data. Thus, these results suggest that the analyzed *H. chilense *germplasm can be divided into two genetically distinct groups.

**Figure 2:**
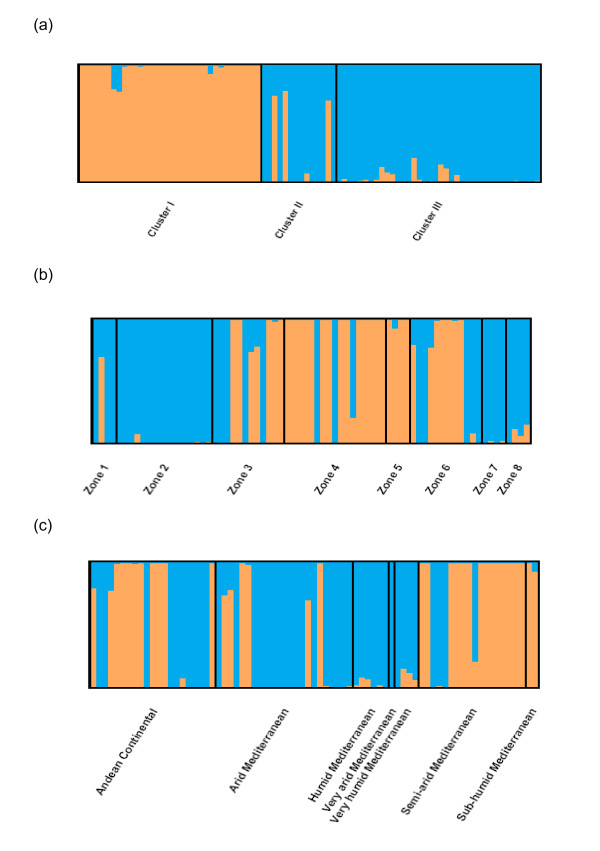
**Membership of *H. chilense *accessions**. Model-based populations when K = 2, and in the predefined groups according to: (a) agro-morphological data; (b) geographic origins; and (c) ecological regions.

To find the key determinants to the inferred structure of these two groups, we investigated the geographical proximity, as well as the morphological, agronomical and ecological characteristics in the accessions belonging to each group. Figure 2a shows the distribution of each accession into the two populations (identified by the Structure software, and designated as group I and group II, hereafter) and the three clusters (according to morphological and agronomical data as classified by Vaz Patto et al. [6]). Geographic origins divided in 8 zones (Figure 2b) and ecological regions (Figure 2c) according to the classification established by DiCastri and Hajek [41] at K = 2 populations, are also shown.

**Figure 3:**
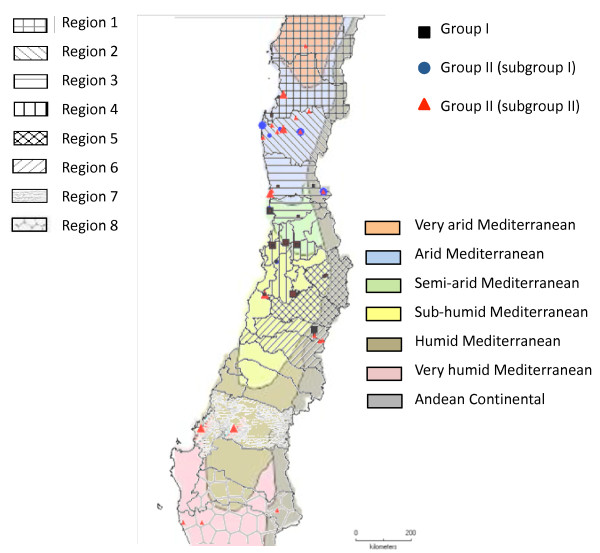
**Geographical and environmental distribution of the *H. chilense *accessions, according to their classification in two populations(group I and group II)**. Symbol size represents more than one sample at the same location.

**Figure 4:**
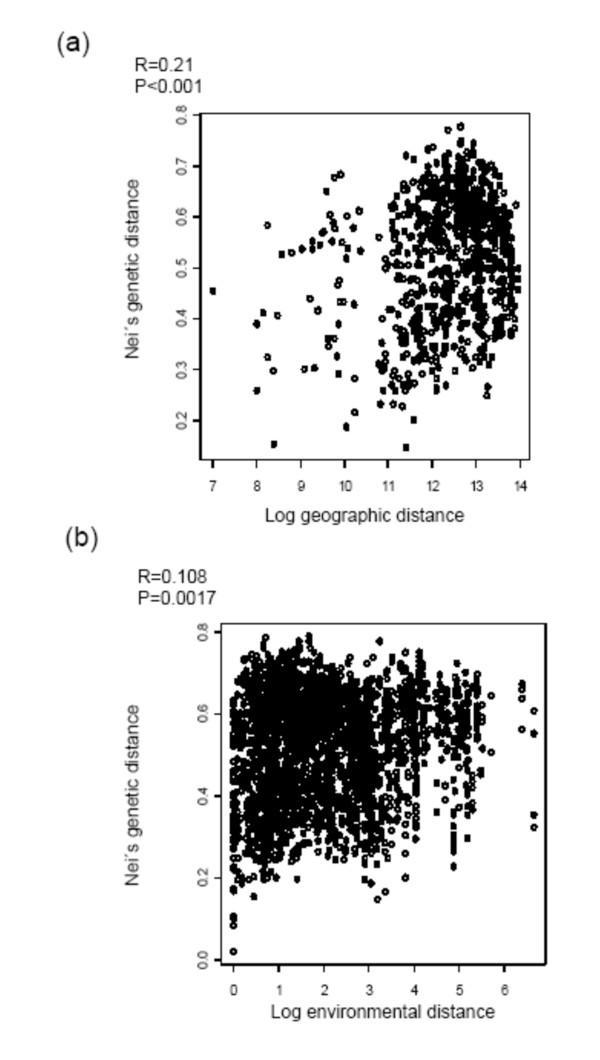
**Mantel test showing the relationship of genetic, geographic and environmental distances**. (4a) Relationship between Nei's genetic distance and Napierian logarithm of geographic distances; and (4b) relationship between Nei's genetic distance and environmental distance.

The genetic structure analysis, according to the geographical origins of the accessions (Figure 2b) revealed that, in some regions, the accessions were grouped according to the geographical location. The zones 2, 4, 5 and 7 showed a uniform structure, while the rest of the zones were more or less admixed. Uniform structure was considered when more than 80% of the accessions in one group had more than 80% of membership in this group. The geographical origin of the accessions and their membership to the inferred groups are represented in Figure 3. We calculated the correlation coefficient (*r*) between the geographic and the genetic distance matrices using the Mantel test [42]. We observed an *r *value of 0.21 (Figure 4a), revealing a low but significant correlation (p < 0.001). The correlation was then analyzed separately for both inferred groups (I and II), and the results showed that group I had a uniform distribution in the Center of the country, yet group II expanded across the North, Center and South of the country. Therefore, group II was analyzed separately for the North, the Center and the South regions. We found a significant correlation between geographical proximity and genetic distance for group I and for group II Central. Thus, our results demonstrate a geographical influence in population structure in the Central Chilean region for both structure groups (additional file 2). Such influence was not detected for the group II accessions, either at the North or South regions (the latter is probably due to the scarcity of accessions).

**Figure 5:**
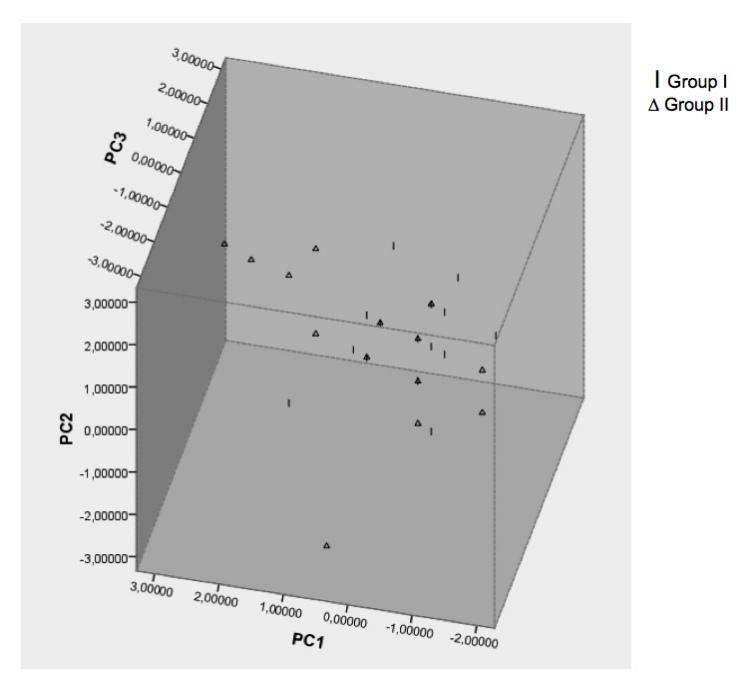
**Principal Component Analysis based on ecogeographic values**. The samples shapes are displayed according to the groups defined by the Structure software.

To investigate the impact of ecological characteristics in the inferred structure (K = 2), the accessions were grouped according to bioclimatic parameters. The results revealed admixed populations, except for some provinces that showed a more uniform structure (like humid Mediterranean, very arid Mediterranean, very humid Mediterranean, and sub-humid Mediterranean; see Figure 2c). Comparative analyses between ecogeographical data (latitude, longitude, altitude, mean temperature of coldest and warmest month, and rainfall of driest and wettest month) and genetic data (Nei's genetic distance matrix) revealed a weak but significant correlation (r = 0.108; p = 0.0017) (Figure 4b). When analyzing the influence of the same ecogeographical data in both structured groups separately, the group I did not show any ecogeographical influence, whereas the group II Central exhibited a significant ecogeographical influence (additional file 3). In addition, the ecogeographical data were used to separate the *H. chilense *accessions through Principal Component (PC) analysis. The first component (PC1, Figure 5 and Table 3) was explained by variation in latitude and rainfall, accounting for 42.9% of the variation. The second component (PC2) accounted for 35.4% of the variation, being explained by variation in temperature and longitude. PC3 accounted for 12.3% of the variation, and was explained by the variation in elevation.

**Table 3:** Principal component analysis based on ecogeographical data of H. chilense accessions.

Variable	Principal Component		
	**PC1**	**PC2**	**PC3**

Elevation	0.126	-0.586	0.755
Warmest month	-0.445	0.779	0.389
Coldest month	-0.568	0.763	0.253
Latitude	0.906	0.327	0.171
Longitude	0.213	0.865	-0.147
Wettest month	0.896	0.181	0.148
Driest month	0.893	0.236	-0.038
Variance (%)	42.9	35.4	12.3

**Figure 6:**
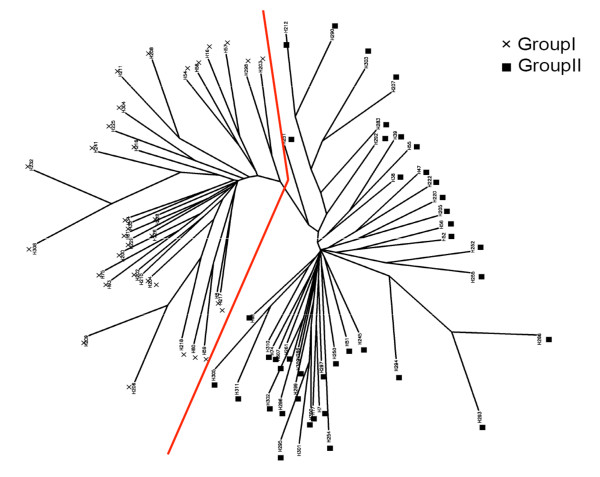
**Unrooted neighbor-joining tree based on the Nei's genetic distances obtained using 49 microsatellites**. The sample shapes indicate the two groups, inferred by the Structure software.

The unrooted Neighbor-Joining (NJ) tree (Figure 6) distinguished two groups of accessions, corresponding to the structure grouping. Neither geographical nor ecological evidence was detected in the grouping. Results of distance and Bayesian cluster analyses evidenced the presence of a structured genetic diversity among the groups. The Analysis of MOlecular VAriance (AMOVA) of the two inferred groups by the Structure software revealed a 33.16% of the genetic variation among groups, with the remaining 66.84% due to differences within groups. The genetic variances within and among groups were significant (F_ST _= 0.331, p < 0.001; being F_ST _the variance among subpopulations relative to the total variance), supporting the presence of a genetic structure.

### Association between markers and ecogeographical factors

**Figure 7:**
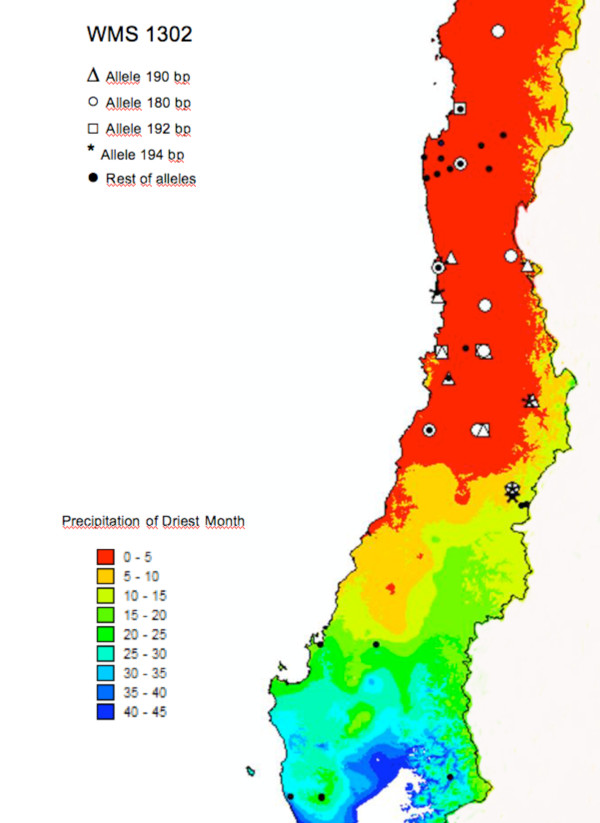
**Distribution of alleles associated with rainfall**. Distribution of alleles 188 (○), 190 (Δ), 192 (□), 194 (*) of the GWM1302 locus associated with rainfall. Non-associated alleles are also shown (●).

We identified 12 outlier loci that detected high or low variability with respect to the expected neutrality. Among those, 11 loci (GBM1350, GBM1064, GBM1008, GBM1060, GBM1464, GWM1047, GBMS14, GWM1302, CFE135, GPW7335, GPW7663 and GPW7577) are candidates for balanced selection, while the locus CFE135 is a candidate for being subjected to positive selection. The 12 markers were assayed for their association with ecogeographical data. The marker GWM1302 exhibited four different alleles (188, 190, 192 and 194 bp) among 10 alleles that were associated with low rainfall (Figure 7). The other markers did not show any significant association with any of the ecogeographical traits.

## Discussion

### Transferability and polymorphism of wheat EST-SSR

The transferability of EST-SSRs across related species has been demonstrated in several species and genera [24,26,29-32,43-45]. Recently, we reported on the usefulness of barley EST-SSRs for genetic analysis in *H. chilense *[33]. In the present work, we show that more than half (54%) of the assayed wheat EST-SSRs can be transferred to *H. chilense*, which is lower than the transferability of the barley EST-SSRs (66%). This is likely due to the fact that wheat is evolutively more distant from *H. chilense *than barley. This result is relatively consistent with the findings of Zhang et al. [30] and Gupta et al. [46], who reported higher transferability of wheat EST-SSRs to barley than to more evolutively distant species, such as maize, rice, sorghum, lolium (ryegrass), oats and purple false brome (*Brachypodium*).

Despite of the evolutive distance, the overall conservation of the wheat EST-SSRs linkage groups in *H. chilense *was high, indicating a good level of synteny between these two species. Only one disagreement was observed, that may be due to chromosomal rearrangements, which may be frequent during speciation [47]. Thus, the wheat EST-SSRs markers transferred to *H. chilense *have an added value as intergeneric syntenic markers, in addition to their direct application to analyze gene diversity. Since numerous additional wheat EST-SSRs are available in the public databases (eg., GrainGenes http://wheat.pw.usda.gov/GG2/index.shtml; [48]), this number could be further increased.

Among the 53 wheat EST-SSRs showing good transferability to *H. chilense*, about 40% exhibited polymorphism between at least two accessions, which represents 20% of the initial set of wheat EST-SSRs. This was lower compared to what was previously observed between wheat and barley (60% of the transferable EST-SSRs; [30]). This is also lower compared to what was found using barley EST-SSRs (36%), because barley is more closely related to *H. chilense *than wheat. Thus, the markers transferred from wheat to *H. chilense *are only those that are more conserved, and therefore likely to show lower polymorphism. Similarly, the number of alleles detected in *H. chilense *with wheat EST-SSRs was lower compared to the number obtained when using barley EST-SSRs (94 vs 162, respectively). On the other hand, due to their wheat origin, they are not suitable for direct in-tube detection methods [49]. Nevertheless these drawbacks are largely overcome by the fact that they provide valuable anchors for synteny inference [50]. Therefore, we conclude that both wheat and barley represent a good source of markers for genetic diversity and structure studies of *H. chilense *germplasm collections. In addition, they represent an invaluable tool for the introgression of *H. chilense *alleles to other cereal species.

### Genetic variability analysis

The microsatellite markers revealed a total of 351 alleles across all the 49 loci. The high level of genetic diversity detected could be an adaptive strategy in response to a heterogeneous environment. According to the marker's origin, the SSRs identified in EST databases detected a lower number of alleles and PIC than those obtained through general genomic libraries, including non-transcribed regions. Such is an expected result, due to the more conserved nature of EST-SSRs. The number of alleles detected and PIC found when using EST-SSRs from wheat was lower than with barley EST-SSR and non-transcribed genomic microsatellites, as expected, due to the evolutionary distance between species. Despite these facts, our results show a sufficient level of variation when using EST-SSRs (both from barley and wheat origins) to carry out genetic structure and future association mapping analysis. Therefore, this is one more case where the EST-SSR markers provide an opportunity to examine the functional diversity of germplasm collections, as shown by Eujayl et al. 2002 [51].

The group I identified by Structure was fixed to one allele in eight loci. Seven of these loci could be assumed as being non-neutral, due to their origin from EST databases. The higher number of fixed alleles in non-neutral SSRs could be explained by temporal variation of external factors, generating selection pressures that maintain variation within populations [52]. This may also be due to the fact that not all nucleotide bases on a transcribed DNA are of selective nature. In fact, the third base of the mRNA triplets is less specific (wobble hypothesis, [53]). Furthermore, some amino acids may share similar chemical properties (eg., nonpolar or polar, including acidic or basic), thus being less prone to generate a phenotypic change. A high number of specific alleles were identified, which could be an indication of the relatively high rate of mutation at SSR loci [54], or to a germplasm with a rich genetic diversity and a divergent population structure. The gene diversity is significantly lower in group I than in group II (Table 2). Therefore, the group II is genetically more diverse, corresponding to accessions present in a wider ecogeographical range.

The Spearman correlation showed that genetic diversity is influenced negatively by altitude and positively by temperature. Accessions in group II showed a higher number of alleles. Besides, they were found mainly in places with low altitude and higher temperature than accessions in group I. Therefore, accessions stressed by cold showed less genetic variation.

In this genetic variability analysis of *H. chilense *germplasm using wheat and barley gSSRs and EST-SSRs, we have defined two main germplasm groups (group I and group II). A previous analysis based on AFLP markers and a Principal-CoOrdinate analysis ([6]) divided the same germplasm into three clusters. The group II defined in this work contains two subgroups, corresponding to the clusters II and III described by Vaz Patto et al. ([6]).

### Genetic structure in *H. chilense*

The analysis of the genetic structure using both Bayesian approaches (Structure and Geneland software) and genetic distance approaches (cluster analysis) of a set of 94 *H. chilense *accessions, using 49 microsatellite markers, revealed two genetically differentiated groups.

The 'admixture model' implemented by Structure gave a better fit to the species ecophysiological clusters, as defined by Vaz Patto et al. [6], and it was chosen for further association analysis. Therefore, the morphological and agronomic characteristics, which determined the ecophysiological clusters, were key determinants of the population structure of the *H. chilense *germplasm. Thus, the two inferred groups are mainly in accordance with the agro-morphological clusters described by Vaz Patto et al. [6], as the group I corresponds to cluster I, while the group II (with the exception of three lines) includes clusters II and III (see Figure 2a). According to the geographical origins and the ecological distribution, the inferred genetic structure showed both uniform and admixed populations (Figure 2b and 2c). The accessions included in the geographical zones 1 and 2 showed a uniform genetic structure. They were found in the driest places of these zones, corresponding with Mediterranean arid environments. The accessions grouped in the geographical regions 7 and 8 revealed also a uniform genetic structure, and were found in the wettest places, corresponding to and Mediterranean humid and very humid environments. This points to an influence of the rainfall in shaping the population structure.

Several studies have been carried out to detect the population structure in barley. In most of them, the key factors affecting the genetic structure were growth habit or spike morphology and geographic origin [15,16,19,20,55-57]. In our study, the morphological and agronomic characteristics also have appeared as the main factors to affect the population structure, although we have also shown that geographic locations and ecological patterns of distribution also affect this structure. The analysis of the total germplasm set revealed low but significant associations between geographical and genetic distance, as well as between ecological and genetic distance. By analyzing separately the three main geographical regions of provenance of the species (North, Center and South), a more significant correlation between geographical and genetic distance was detected for the accessions from Central Chile, but no association was found either in Northern or in Southern Chile accessions. This is in agreement with the basal phylogenetic position of *H. chilense *in South America, established initially in Central Chile from a long-distance continental dispersal from North America, followed by two independent dispersals to the North and to the South [58]. On the other hand, by analyzing both population structure groups separately, a higher correlation between ecological and genetic distance could be detected for group II accessions, but no correlation was found for group I accessions. In fact, group II accessions have shown a better ability to colonize the North and the South Chilean regions (see Figure 3). Thus, the adaptation to geographic and ecological factors may be one of the causes of the genetic structure in the studied germplasm. Thus, the results of our work illustrate the interest to further investigate how morphological characteristics and ecophysiological traits affect the species selection and the population structure. Moreover, the presence of a high level of structure within the *H. chilense *germplasm should be considered in future association mapping studies. The AMOVA detected higher differences among individuals within-population structure groups than among groups, which is consistent with findings from other studies, indicating that considerable genetic diversity is partitioned within rather than between wild barley populations [59-63]. The proportion of genetic variation within population groups reflected high levels of genetic diversity.

### Association of markers with ecogeographical factors

The loci that show unusually low or high levels of genetic differentiation are often assumed to be under natural selection [64]. The accessions that show association of alleles of the locus GWM1302 with low precipitation belong mainly to the group I, and were collected from dry places, thereby suggesting that this environmental factor is involved in a local adaptation after colonization.

Significant correlations between microsatellite markers and ecogeographical factors have been observed in several studies in wild wheat [65] and in wild barley [66], suggesting the impact of natural selection on these markers by creating regional divergence.

Genetic clustering in a principal component analysis revealed that the combination of geographic and ecological data, such as the latitude with rainfall, as the main contributor to the genetic structure of the *H. chilense *germplasm. The second principal component explained by longitude and temperature significantly contributed to the separation of the two groups. Hübner et al. [21] studied the population structure in *Hordeum spontaneum *and found a strong correlation of population structure with temperature and precipitation. In our study, the genetic structure of the analyzed germplasm showed a correlation with morphological and ecophysiological characteristics, influenced also to a minor extent by geographic and ecological factors.

## Conclusions

Our study shows the utility of barley EST-SSR for the genetic analysis of *H. chilense*, with a remarkably high level of polymorphism within this species, despite of the evolutionary distance between the wheat and barley genera. The current set of SSR markers available for *Hordeum chilense*, which includes wheat and barley gSSRs and EST-SSRs, is useful to analyze the genetic structure and ecogeographical adaptation of *H. chilense *wild barley populations. Both wheat and barley represent a good source of markers for genetic diversity and structure studies of *H. chilense *germplasm collections. In addition, they represent an invaluable tool for the introgression of *H. chilense *alleles into other cereal species, and are useful as anchors for the syntenic maps. The analyzed germplasm can be divided into two groups, with morphological and ecophysiological characteristics being key determinants of the population structure. Geographic and ecological structuring was also revealed in the analyzed germplasm. A significant correlation between geographical and genetic distance was detected in the Central Chilean region for the first time in the species. In addition, significant ecological influence in genetic distance has been detected for one of the population structure groups (group II) in the Central Chilean region. Finally, one marker was found significantly associated with precipitation. These findings have a potential application in cereal breeding.

## Methods

### Plant material and DNA sampling

The DNA collection, isolated by Castillo et al. [38], consisted of 94 samples of *H. chilense *collected during several expeditions to Chile [67,68]. This germplasm is maintained at the Germplasm Bank of the Institute for Sustainable Agriculture (Prof. A. Martín, IAS, CSIC, Cordoba, Spain). Eight samples of *H. chilense *DNA were used for selecting transferable and polymorphic microsatellite markers, using *Triticum aestivum *cv. 'Chinese Spring' DNA as control, to corroborate the pattern of the microsatellites and the fragment sizes. In addition, a DNA set of *T. aestivum*-*H. chilense *addition lines developed by Miller [37] were used to determine the chromosomal locations of the polymorphic SSR. The addition lines for chromosomes 4H^ch^, 5H^ch^, 6H^ch ^and 7H^ch ^were disomic, whereas the addition lines for chromosomes 1H^ch^S, 2H^ch ^alpha arm, 5H^ch^, 6H^ch^S arm and 7H^ch ^alpha and beta arm were ditelosomic.

### Amplification and transferability of wheat EST-SSRs

A selection of 98 SSRs derived from wheat ESTs [69,70] uniformly distributed across wheat chromosomes were initially screened for their transferability and polymorphism in *H. chilense *genome, and 20 were selected for germplasm analysis. A set of 21 barley EST-SSRs and eight wheat and barley g-SSRs, previously identified as useful for the genetic analysis in *H. chilense *[33,68] was added. In total, 49 polymorphic microsatellite markers were applied for fingerprinting *H. chilense *accessions. The polymerase chain reaction (PCR) amplification and fragment analysis were as previously described [43,71,72], or PCR was carried out using the M13 protocol as described in Nicot et al. [69], with an annealing temperature of 60°C for 30 cycles (30 s at 94°C, 30 s at 60°C, and 30 s at 72°C) and 56°C annealing for eight cycles. Amplification products were visualized using an ABI PRISM 3700 Genetic Analyzer from Life Technologies (Carlsbad, CA, USA). The fragment sizes were calculated using GeneMapper software from the same manufacturer.

### Ecogeographical data

The geographic data (altitude, latitude and longitude) of 76 accessions were available [6], and thus they were used to project the data using the DIVA-GIS software http://www.diva-gis.org;. The geographic location of the study area was between 28°15' and 38°42' South latitude and between 70°18' and 73°24' West longitude. The altitude on the sites varied within a wide range, from sea level to high mountains (> 2000 m). Since only one accession is available in some provinces, the accessions were grouped in eight zones along Chile, from North to South, including in some cases various close provinces with similar ecological characteristics. The ecological data like rainfall of wettest and driest month and mean temperature of the warmest and coldest month were obtained for each site using DIVA-GIS. The ecological regions were described following the bioclimatic classification of DiCastri and Hajek [41].

### Statistical analysis

The summary statistics including the number of alleles per locus, polymorphism information content (PIC) values and gene diversity were determined using the application PowerMarker version 3.25 [73]. The unrooted neighbor-joining (NJ) tree was constructed using the Nei's index distance [74]. One thousand matrices were obtained by bootstrapping, and the consensus tree was constructed with the program Consense of the Phylip package (version 3.66) [75]. The dendrogram was visualized using the TreeView 1.6.6 software [76]. We performed a Mantel test correlation [42] between Nei's genetic distance and the natural (Napierian) logarithm of the geographic distances, using the library ade4 in the R package (version 2.10.1; R Development Core Team 2008) [78].

A Bayesian model-based analysis for inference of population structure was performed using the program Structure (version 2.2) [78] to estimate the number of groups (K) represented by all sampled individuals and the individual admixture proportions. The Structure software assumes a model in which there are K populations (where K may be unknown), each being characterized by a set of allele frequencies at each locus. Individuals in the sample are probabilistically assigned to a particular population, or associated to two or more populations (if their genotypes indicate that they are admixed). The number of clusters was inferred using 20 independent runs with 100,000 burn-ins and 100,000 iterations after burn-ins, following the admixture ancestry model and correlated allele frequencies, with K ranging from 1 to 10. We have followed the procedure by Evanno et al. [39] to better detect the real number of clusters determined by Structure. Also, the clusteredness index [40] was calculated, which is based on the Q matrix of Structure, being 1 when individuals are assigned completely to a single cluster and 0 when they are equally assigned to all clusters. The individuals can have membership coefficients summing 1 across clusters.

The Distruct 1.1 software [40] was used to graphically represent the estimated population structure, according to geographic proximity, ecological region and agronomical data. Each individual was represented by a thick line, which was partitioned into K colored segments, representing the individual's estimated membership fractions in K clusters.

The genetic structure of the population was also inferred by the Geneland package [79], implemented in the R software. The Geneland software uses geographic coordinates and does not assume admixture, whereas the Structure software does not use geographic coordinates and does assume admixture. We carried out five independent runs using independent allele frequencies with 100,000 iterations, from which each 100th observation was sampled from the Markov chain, with minimum and maximum K being 1 to 10. The run with the highest likelihood was post-processed to obtain the posterior mode of population membership. The genetic differentiation among genetic groups inferred by Structure was estimated by hierarchical analysis of molecular variance (AMOVA), implemented in the Arlequin 3.0 software [80].

We used the Lositan software [81] to identify outlier loci that had excessively high or low Fst compared to neutral expectations. The basic rationale is that (i) loci influenced by directional (also called adaptive or positive) selection will show a larger genetic differentiation than neutral loci; and that (ii) loci that have been subject to balancing (also called negative or purifying) selection will show a lower genetic differentiation. Thus, the methods generally consist of identifying loci that present Fst coefficients that are "significantly" different from those expected under neutral theory (they are called outlier loci). To avoid false positives caused by population structure, the Fst was calculated for the inferred structure groups (the significance level chosen was 0.001, which corresponds to a statistical significance level of 0.05), applying a Bonferroni standard correction. The association of alleles of outlier loci with ecogeographical factors was assayed by linear regression analyses, using the SPSS package version 17.0.0 from SPSS (Chicago, IL, USA). Alleles with frequencies below 5% were excluded. Alleles of each locus were introduced as dependent variables in the model and ecogeographical factors were the independent variables. Significance was calculated for the model, which included only one allele, with the significance threshold set at 0.05, using a Bonferroni correction, as already mentioned.

Values of environmental variables were first standardized and the Euclidean distance between the samples was computed using SPSS. The correlation between genetic distance and environmental distance in the collection was calculated by the Mantel test. Also, the Principal Component Analysis (PCA) was computed from environmental values, and the samples were plotted in genetic structure grouping. The Spearman rank correlation was used to assess differences in mean number of alleles and ecogeographic variables among the inferred groups.

## Authors' contributions

AC carried out the experiments and drafted the manuscript. CF, PS, GD and PH were involved in designing and planning the work, interpreting the results and critically editing the manuscript. PH, CF and PS conceived the study. PH coordinated the study and helped to draft the manuscript. All authors have read and approved the final manuscript.

## Supplementary Material

Additional file 1 **Estimated number of populations from Geneland analysis**. (a) Posterior density distribution of the number of clusters estimated from analysis in five replicates; and (b) genetic assignment of *H. chilense *individuals. Click here for file

Additional file 2 **Mantel test showing the relationship between genetic distance and geographic distance**. (B1) Relationship between Nei's genetic distance and Napierian logarithm of geographic distances for group I accessions; and (B2) relationship between Nei's genetic distance and Napierian logarithm of geographic distances for group II-Center accessions. Click here for file

Additional file 3 **Mantel test showing the relationship between genetic distance and environmental distance for group II accessions**. Plot of genetic distance *vs*. Ln (environmental distance). Click here for file
