# Supplementary material for: Genetic structure and ecogeographical adaptation in wild barley (Hordeum chilense Roemer et Schultes) as revealed by microsatellite markers
Source: BMC Plant Biol. 2010 Nov 30;10:266. doi: 10.1186/1471-2229-10-266 (PMC3014967; doi:10.1186/1471-2229-10-266)

Estimated number of populations from Geneland analysis. (a) Posterior density distribution of the number of clusters estimated from analysis in five replicates; and (b) genetic assignment of *H. chilense* individuals.

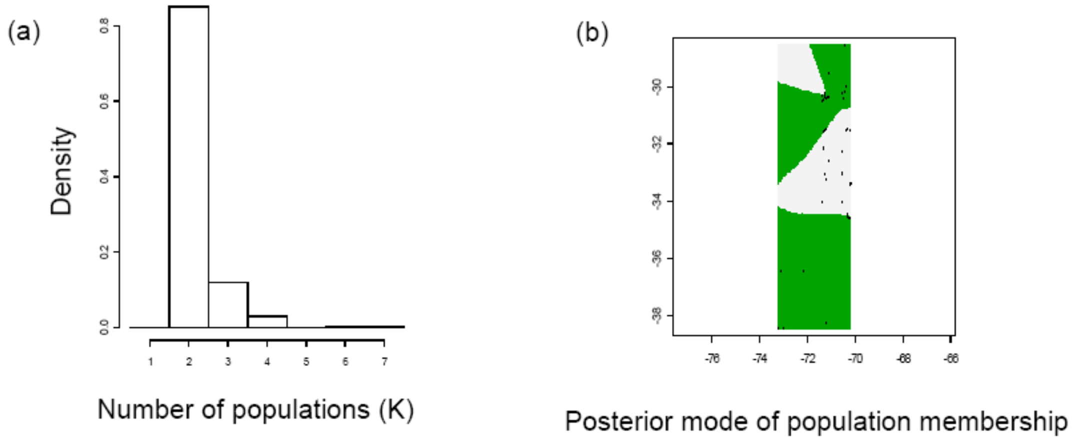

Supplement: Additional file 1 — Estimated number of populations from Geneland analysis. (a) Posterior density distribution of the number of clusters estimated from analysis in five replicates; and (b) genetic assignment of H. chilense individuals. [file 1471-2229-10-266-S1.PDF]
