# Supplementary material for: Genetic structure and ecogeographical adaptation in wild barley (Hordeum chilense Roemer et Schultes) as revealed by microsatellite markers
Source: BMC Plant Biol. 2010 Nov 30;10:266. doi: 10.1186/1471-2229-10-266 (PMC3014967; doi:10.1186/1471-2229-10-266)

Mantel test showing the relationship between genetic distance and geographic distance. (B1) Relationship between Nei's genetic distance and Napierian logarithm of geographic distances for group I accessions; and (B2) relationship between Nei's genetic distance and Napierian logarithm of geographic distances for group II-Center accessions.

B1. Group I

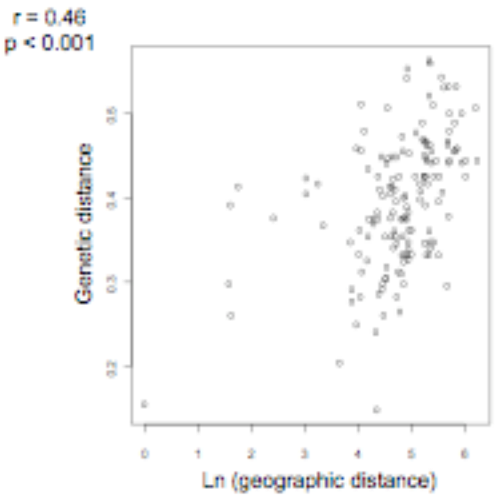

B2. Group II-Center

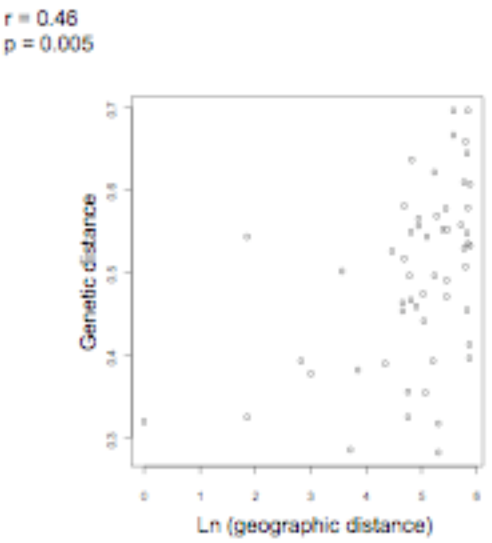

Supplement: Additional file 2 — Mantel test showing the relationship between genetic distance and geographic distance. (B1) Relationship between Nei's genetic distance and Napierian logarithm of geographic distances for group I accessions; and (B2) relationship between Nei's genetic distance and Napierian logarithm of geographic distances for group II-Center accessions. [file 1471-2229-10-266-S2.PDF]
