# Supplementary material for: Genetic structure and ecogeographical adaptation in wild barley (Hordeum chilense Roemer et Schultes) as revealed by microsatellite markers
Source: BMC Plant Biol. 2010 Nov 30;10:266. doi: 10.1186/1471-2229-10-266 (PMC3014967; doi:10.1186/1471-2229-10-266)

Mantel test showing the relationship between genetic distance and environmental distance for group II accessions.

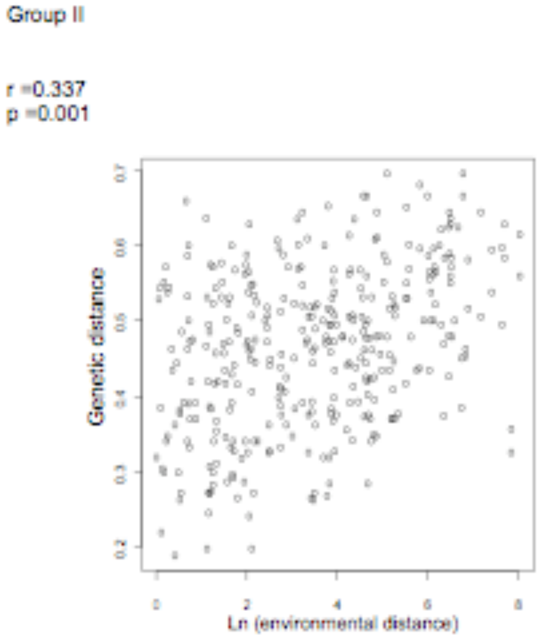

Supplement: Additional file 3 — Mantel test showing the relationship between genetic distance and environmental distance for group II accessions. Plot of genetic distance vs. Ln (environmental distance). [file 1471-2229-10-266-S3.PDF]
